# Connectedness to Nature Does Not Explain the Variation in Physical Activity and Body Composition in Adults and Older People

**DOI:** 10.3390/ijerph182211951

**Published:** 2021-11-14

**Authors:** Andreia Teixeira, Ronaldo Gabriel, José Martinho, Graça Pinto, Luís Quaresma, Aurélio Faria, Irene Oliveira, Helena Moreira

**Affiliations:** 1 University of Trás-os-Montes and Alto Douro, 5000-801 Vila Real, Portugal; 2 Centre for the Research and Technology of Agro-Environmental and Biological Sciences (CITAB), Department of Sports Science, Exercise and Health, University of Trás-os-Montes and Alto Douro, 5000-801 Vila Real, Portugal; rgabriel@utad.pt; 3 Geosciences Centre (CGeo), Department of Geology, University of Trás-os-Montes and Alto Douro, 5000-801 Vila Real, Portugal; martinho@utad.pt; 4 Center in Sports Sciences, Health Sciences and Human Development (CIDESD), Department of Sports Science, Exercise and Health, University of Trás-os-Montes and Alto Douro, 5000-801 Vila Real, Portugal; gpinto@utad.pt; 5 Department of Sports Science, Exercise and Health, University of Trás-os-Montes and Alto Douro, 5000-801 Vila Real, Portugal; luisquar@utad.pt; 6 Center in Sports Sciences, Health Sciences and Human Development (CIDESD), Department of Sport Science, University of Beira Interior, 6201-001 Covilhã, Portugal; afaria@ubi.pt; 7 Centre for the Research and Technology of Agro-Environmental and Biological Sciences (CITAB), Department of Mathematics, University of Trás-os-Montes and Alto Douro, 5000-801 Vila Real, Portugal; ioliveir@utad.pt; 8 Center for Computational and Stochastic Mathematics, CEMAT-IST-UL, University of Lisbon, 1600-214 Lisbon, Portugal; 9 Research Center in Sports Sciences, Health Sciences and Human Development (CIDESD), Department of Sports, Exercise and Health Sciences, University of Trás-os-Montes and Alto Douro, 5000-801 Vila Real, Portugal; 10 Centre for the Research and Technology of Agro-Environmental and Biological Sciences (CITAB), Department of Sports, Exercise and Health Sciences, University of Trás-os-Montes and Alto Douro, 5000-801 Vila Real, Portugal

**Keywords:** nature relatedness, gender, abdominal adiposity, muscle condition, demographic factors, accelerometer

## Abstract

Connectedness to nature (CN) is a significant predictor of pro-environmental behaviours, human health and well-being. However, research on how this connection to the natural world might promote a more active lifestyle and improve body mass composition according to gender is lacking. This study investigated the influence of CN on physical activity (PA) and body composition in adults and older people. We recruited a sample of 219 individuals (77 men and 142 women), and a self-administered questionnaire was used to measure CN and obtain demographic data. Body composition was assessed by bioimpedance, and PA was assessed by accelerometry. Correlations and stepwise multiple regressions were used in data analysis. CN’s association with other variables was more pronounced in women than in men, and we only identified significant associations with steps/day and body composition. However, this variable would not be included in the regression models that we developed. Adiposity levels and muscle status were significant predictors of PA in women. In both genders, age, percentage of fat mass and fat-free mass were selected as regressors in the models developed for visceral fat area and muscle condition (R^2^ Adjusted ≥ 0.908).

## 1. Introduction

### Highlights

- Connection to nature was associated with central adiposity and muscle condition in women.
- Women who are more connected to nature perform a greater number of daily steps.
- Connection to nature is higher among older people and employees.
- Connection to nature did not explain variation in physical activity and body composition.

The relationship between people and nature received growing attention [1] from researchers, health professionals and environmentalists due to its effect on human and environmental well-being. The concept of connectedness to nature (CN) seeks to describe the feeling of an affective (emotional) and cognitive bond with the natural environment and the sense of a person’s place in nature [2].

In this respect, research on CN in recent years has focused substantially on the benefits of this connection for psychological well-being. People who are more connected to nature are more satisfied with life [2] and have reported greater happiness [3,4,5] and positive affects [6,7]. CN is also associated with social well-being (e.g., social acceptance) [8], mindfulness [9], meaning of life and vitality [10,11]. Other studies have also reported that a stronger relationship with nature increases self-esteem [12,13] and reduces anxiety and depression [14,15,16]. 

Beyond this, recent systematic reviews [17,18] have suggested that feelings of connection and restoration from nature are linked with the adoption of pro-environmental behaviours, indicating that individuals who are more connected with nature are more likely to act in an environmentally friendly manner than those who feel less connected.

This connection with the natural environment has become increasingly important and is now more pronounced due to the pandemic, which has necessitated many changes in people’s lifestyles, norms and attitudes (e.g., the adoption of remote working practices, the establishment of regular exercise habits in urban parks and increased awareness of the importance of experiences in nature) [19]. According to Robinson et al. [20], people reportedly spent significantly more time in nature and visited natural spaces more often during the pandemic, and this contact may contribute to higher levels of physical activity (PA).

Despite the extensive research into this affective and experiential relationship with nature, there remain significant gaps. First, the analysis of gender differences in this construct is still lacking [21]. Recognising that there are differences between genders when studying emotions [22] and that connection with nature has an emotional component, it seemed interesting to develop separate analyses according to gender. Second, there are still limited studies that examine the influence of demographic characteristics on the connection with nature in adults and older people [23]. Third, when investigating the relationship between PA (indoor and outdoor) and CN [15,24,25], the first variable is usually self-reported, conferring a limited reliability of the results. According to Skender et al. [26], when assessed by a questionnaire, PA tends to be overestimated, and the use of motion sensors, such as pedometers and accelerometers, represents more accurate methods of assessing PA levels. Lastly, the hypothesis that the connection with the natural environment may have an impact on body composition has not yet been explored. Examining this aspect of health is particularly relevant for governments, organisations and communities when developing integrated policies and practices that promote this connection to the natural environment, allowing for synergistic improvements in public and environmental health [27].

Thus, this study aimed to contribute to this growing area of research by exploring the relationship between CN and PA and body composition as well as analysing the predictors that explain the variation in these health-related variables in men and women.

## 2. Materials and Methods

### 2.1. Study Location

This study was undertaken in Vila Real, a city located in the north of Portugal. Approximately 90% of our sample belonged to 16 parishes of this municipality [28], covering 222.57 km^2^, with a human population of approximately 44,644 people. These parishes benefit from 19,204 ha of green space, with a ratio of 8275 m^2^ of green space [29] per inhabitant (Figure S1).

### 2.2. Ethics Statement

This research was conducted with the approval of the Ethics Committee of the University of Trás-os-Montes and Alto Douro (Ref: Doc51A-CE-UTAD-2020), conformed to the recommendations of the Declaration of Helsinki, and respected the measures for preventing the transmission of SARS-CoV-2 infection. Participants were fully informed of the purpose, benefits and risks of the study.

### 2.3. Study Design and Participants

We conducted a cross-sectional study in winter between December 2020 and February 2021, with maximum and minimum temperature values of 10.6 °C and 3.0 °C, respectively [30]. Participants were recruited from university settings and the community via oral invitations, social media and email listservs. However, participation was strongly determined by the results being individually clarified to each participant at the time of the evaluation and sent in writing, which was reflected in the word-of-mouth advertising. A specific recruitment goal was to enrol equal numbers of men and women in the study sample. Nevertheless, because we entered lockdown, we had to cancel the previously arranged evaluations with male subjects. The sample consisted of 219 individuals, including 77 men (41.62 ± 15.12 years) and 142 women (40.10 ± 15.49 years), aged 18 to 75 years. The eligibility criteria included (1) age ≥ 18 years; (2) ability to read and understand Portuguese; (3) willingness to wear an activity-monitoring device on the wrist for four consecutive days; (4) non-use of a pacemaker. Participants who did not complete all evaluations or did not use the accelerometer for a minimum of ten hours per day on four assessment days were excluded from the sample. The evaluators were trained both technically and scientifically.

### 2.4. Measures

Green space data: Green space was quantified using the Land Use Regime Map, containing the following 10 land cover classes: (1) agricultural area; (2) cultural area; (3) economic activities area; (4) area for special use—equipment and infrastructure; (5) agro-forestry; (6) forest; (7) natural space; (8) equipment and infrastructure area; (9) residential area; (10) green space. According to the adopted definition of green space—open, accessible and available space that may include agricultural, forest, grassland or other natural areas of at least 1 ha [31,32]—classes 1, 5, 6, 7 and 10 were considered.

Connectedness to Nature Scale (CNS): The CNS was designed to explicitly measure the degree to which a person feels emotionally connected to nature [2]. As reported by Whitburn, Linklater and Abrahamse [18], this is a widely used scale in the literature and has been adapted and validated for several languages. In our study, we used the Portuguese version of the CNS [33]. The scale is composed of 14 items, with response options on a 5-point Likert scale ranging from 1 (strongly disagree) to 5 (strongly agree), where three items were reverse-scored (numbers 4, 12 and 14). Final scores of 4 and 5 points were indicative, respectively, of a high and a very high relationship with nature. The scale was previously demonstrated to have a Cronbach alpha of 0.84 [2]. In the current sample, the alpha was 0.7, indicating acceptable reliability.

Contexts for PA: Based on the options provided by the Eurobarometer [34], participants selected one of the following PA contexts: (a) in parks, outdoors, etc.; (b) at home; (c) on the way to home, school, work or shop; (d) at a health or fitness centre; (e) at a sports centre; (f) at work; (g) at school or university; (h) elsewhere (spontaneous); (i) don’t know.

Objectively measured PA: Accelerometery is the most commonly used and objective method in clinical and epidemiological research [35] and is applied to different populations. In our study, each participant wore a triaxial accelerometer wGT3X-BT (Actigraph Inc., Pensacola, FL, USA) on the non-dominant wrist and was instructed to only remove the device for bathing or performing any aquatic or other activity that compromised the integrity of the equipment and/or the safety of the individual. All participants who agreed to wear the accelerometer were given verbal and written instructions on their use immediately before commencing the 4 day study period (two weekdays and two weekend days). In addition, a record sheet was provided to register the times of placement and removal of the accelerometer. A valid wear day consisted of at least 10 h of wear time [36]. The start of the device was programmed for 6:00 a.m. on the first day of evaluation, and PA records were considered in 15 s periods. Accelerometers were initialised to capture and store the accelerations at 100 Hz. ActiGraph software support (ActiLife, v6.13.3, Pensacola, CA, USA) was used to initiate, download and process the data. The accelerometers were calibrated according to the age, gender and weight of the user, and the output was expressed as counts per minute. Non-wear time was defined as 90 consecutive minutes of zero counts, with an allowance of 2 min of non-zero counts, provided that there were 30 min consecutive zero count windows up- and downstream [37]. The variables measured by accelerometery were as follows: total PA (TPA, min/week); moderate-vigorous PA (MVPA, min/week) and steps/day (No.). A cut-off point of 4836 counts per minute was used to assess minutes in moderate-vigorous PA [38]. The cut-off point for MVPA was 150 min/week [39].

Anthropometry and body composition: Body height (BH) was measured with a stadiometer SECA 220 (Seca Corporation, Hamburg, Germany), considering a tolerance threshold of 2 mm [40]. We used the octopolar bioimpedance InBody 720 (Biospace, Seoul, Korea) to separately measure the impedance of the subject’s trunk, arms and legs at six different frequencies for each of the body segments and the reactance at three frequencies [41]. Participants were instructed to (1) avoid food or drink intake for at least 4 h; (2) not perform MVPA 12 h before the evaluation; (3) use the bathroom 30 min before the test (to reduce the volume of urine and faeces); (4) avoid consumption of alcoholic beverages for at least 48 h; (5) not wear metal jewellery; (6) not perform the test one week before the onset of menstruation and during the menstrual period (only applicable to women up to menopause) [41].

We measured the following variables: body mass (BM, kg); body mass index (BMI, kg/m^2^); fat mass (FM, kg and %); visceral fat area (VFA, cm^2^); fat-free mass (FFM, kg); skeletal muscle mass (SMM, kg); appendicular skeletal muscle mass (ASMM, kg); appendicular skeletal muscle mass index (ASMMI = ASMM/BH^2^, kg/m^2^); trunk skeletal muscle mass (TSMM, kg); trunk skeletal muscle mass index (TSMMI = TSMM/BH^2^, kg/m^2^). The evaluation of VFA is described in the user manual [41], which defines this parameter as the area of transversal cut in the abdominal zone (L4–L5). The data were electronically imported into spreadsheets using the Lookin’Body 120 software. Low muscle mass was defined as ASMMI < 7.0 kg/m^2^ in men and <5.5 kg/m^2^ in women [42]. The cut-off points for elevated visceral fat area and obesity were as follows: VFA ≥ 100 cm^2^ [43]; FM ≥ 25% in men and FM ≥ 32% in women [44].

Demographic data: We collected information regarding marital status, number of children, education, occupational status, residence place, car ownership and dog ownership. The details of the operationalisation of these variables are provided in the Supplementary Materials, Table S1.

### 2.5. Procedures

All participants were individually tested in the Laboratory of Biomechanics, Body Composition and Health (Lab2Health) of the University of Trás-os-Montes and Alto Douro. First, the participants provided written consent to participate in the study. Each participant completed a questionnaire consisting of the previously described CNS and questions related to demographic variables. After that, the body composition was evaluated by bioimpedance, followed by the delivery of the accelerometer. The evaluations in the laboratory took approximately 30 min.

### 2.6. Statistical Methods

Data analyses were performed using SPSS 27.0 package (SPSS Inc., Chicago, IL, USA) and a *p*-value of ≤0.05, which was considered for statistical significance. Continuous data were expressed as mean ± standard deviation, and qualitative variables were presented as absolute frequencies and percentages. The normality of the samples was analysed using the Kolmogorov–Smirnov test. For comparison of means between groups, we used the Student’s *t*-test for numerical variables with a symmetrical distribution, or the Mann–Whitney test for data for numerical variables with an asymmetric distribution and for ordinal variables. The associations between variables were determined using the Pearson correlation coefficient for continuous scale variables when data are normally assumed, Spearman correlation when at least one variable is ordinal or when scale data are non- normal, and point–biserial correlation when one variable is continuous and the other has two categories coded as 0 and 1 [45]. For an independent variable that is categorical and a dependent variable that is at the scale or interval level, Eta value was obtained and used as a measure of association. The square of Eta is interpreted as the proportion of variation in the dependent variable, which is explained by the independent variable [46]. Furthermore, a stepwise regression analysis was used to examine the factors that explained the variation in PA and body composition. Since only numerical variables are allowed to build predictive models in multiple linear regressions, categorical predictors, including nominal and ordinal variables, were converted to binary code using dummy variables before modelling. The inclusion of different variables in the model was performed, considering that the different interactions between variables were found to be statistically significant in the univariate analysis. Multicollinearity was rejected for tolerance > 0.1, variance inflation factor < 10, condition index < 30 and variance proportion < 90%. In some cases, transformations of the variables were performed to normalize the distribution.

## 3. Results

### 3.1. Data Description

Participants’ mean age was 40.63 ± 15.35 years old, and 64.8% of the sample was female. We distinguished statistically significant gender differences for all body composition variables, except for VFA (*p* = 0.54). We observed obesity in 22.1% of men and 46.5% of women, with over 57% of the sample revealing high levels of intra-abdominal adiposity. Based on the ASMMI values, we identified low muscle mass in 13 women and one man. Regarding PA, no gender differences were observed, and the MVPA levels of the sample were 179.13 ± 114.36 min/week. One-half of the women and 46.8% of the men showed PA levels below those recommended in the literature. We observed no gender differences in CN (*p* = 0.59), and the mean value of the sample was 3.74 ± 0.43 points (Table 1).

Most participants were married/cohabiting (48.4%) and had at least one child (53%). Regarding education, 39.3% of individuals reported having high school education, followed by 32.9% reporting having a post-graduate/master’s/doctoral degree. More than half of the sample (67.1%) was employed. Concerning the residence place, 56.2% of the respondents lived in urban areas, 89% had access to or owned a car, and 32.4% were dog owners. The privileged contexts for PA were parks and outdoor spaces (Table 2).

### 3.2. Relationships between Variables

In both genders, age, marital status and occupational status showed a significant (*p* ≤ 0.05) association with the CN (Table 3). Men who were dog owners and women with more children documented a higher CN (r = 0.329 and r = 0.235, respectively, *p* ≤ 0.01). Only in women, the CN exhibited a significant correlation (*p* ≤ 0.05) with steps/day (r = 0.209) as well with some variables of body composition, particularly VFA (r = 0.274, *p* ≤ 0.01) and ASMM (r = −0.187, *p* ≤ 0.05).

Women with higher central adiposity and better regional muscle condition documented a higher number of daily steps (Table 4). A significant relationship (*p* ≤ 0.01) was also identified between the latter variable and age (r = 0.337) as well as occupational status (η^2^ = 0.362) (Table S2). Associations were also identified in the female gender (*p* ≤ 0.05) between contexts for PA practice and VFA (η^2^ = 0.296), as well as for marital status (η^2^ = 0.272).

In men, the contexts for PA practice revealed an association (*p* ≤ 0.05) with educational level (V = 0.321), with age and TSMMI (η^2^ = 0.405 and η^2^ = 0.441, respectively) (Table 4 and Table S2).

The intra-abdominal adiposity in women showed a significant association (*p* ≤ 0.01) with relative fat mass (r = 0.809), age (r = 0.790) and number of children (r = 0.541) (Tables S2 and S3). The presence of higher MVPA levels and lower adiposity favoured the muscle condition of these women. Higher FFM has been shown to be associated with better total and regional muscle condition in females (*p* ≤ 0.01), with SMM also influenced by education (r*_s_* = 0.214, *p* ≤ 0.05) and by age (r = −0.218, *p* ≤ 0.01).

The presence of higher adiposity levels in men compromised SMM (r = −0.227, *p* ≤ 0.05) and intra-abdominal adiposity (r = 0.919, *p* ≤ 0.01), with the latter being amplified (*p* ≤ 0.01) by age (Tables S2 and S3). The FFM revealed very high levels of association (*p* ≤ 0.01) with SMM and ASMM, with an inverse relationship being seen between this last variable and age (r = −0.347).

### 3.3. Multivariate Linear Regression Models

Despite the significant association of CN with steps/day in women, the first was excluded from the model as it did not reveal any explanatory power (Table 5).

In these, the %FM explained (SEE = 0.307 min/week) the variation of MVPA in only 7.4%. The parameters estimated in the model developed for women’s steps/day included (*p* ≤ 0.01) age (β = 0.544), VFA (β = −0.365) and TSMMI (β = 0.345). In men, the occupational status (β = 0.332) was the only predictor selected in the model, presenting an Adjusted R^2^ of 9.8%.

Concerning the regressions that were developed for some variables of body composition (Table 6), only in women was the CN considered in the estimation of the model parameter developed for the VFA and ASMM. However, this variable was excluded, as it did not reveal a significant explanatory power.

In the stepwise regression models developed for the VFA, both genders showed high adjusted R^2^ values (≥91%), in which %FM and age were positive and significant predictors (*p* ≤ 0.01). The variation in VFA was further explained by occupational status (β = 0.081, *p* = 0.012) and PA contexts (β = 0.059, *p* = 0.023) in women and by number of children (β = 0.133, *p* = 0.02) in men.

The variation in SMM, in both genders, was explained by FFM and %FM (β = 0.993 and β = −0.020 for men and β = 0.990 and β = −0.016 for women), where the Adjusted R^2^ values were higher than 99%. In women, higher MVPA levels (β = 0.016, *p* ≤ 0.01) also contributed to better muscle condition.

In men and women, age and FFM proved to be significant predictors of ASMM, with adjusted R^2^ values ≥ 0.930.

## 4. Discussion

The present study aimed to analyse, for each gender, the influence of CN on PA levels and body composition in adults and older people. In all the developed regression models, CN was not shown to be a significant predictor.

The parameters estimated in the models developed for the body composition variables included %FM, FFM and age, and for VFA, some demographic parameters were also introduced in the models (occupational status and contexts for PA for women and number of children for men). They presented adjusted R^2^ values higher than 0.91. Regarding PA and with respect to the male gender, only occupational status proved to be a significant predictor of steps/day. In the case of women, variables related to adiposity and regional muscle condition were included as regressors.

### 4.1. Connectedness to Nature and Demographic Variables

Gender: As in other studies [2,21,47], we observed no differences in CN between genders. However, some authors have indicated that CN tends to be higher in women [8,10,13,48,49], since they usually exhibit a higher number of pro-environmental behaviours compared to men [49]. The fact that the sample lives in an area with a high availability of green space (8275 m^2^ per inhabitant) may have influenced these results, given that they have the same opportunities to connect with natural spaces. Safe and inclusive access to these spaces, particularly for women, older adults and people with disabilities, is very important to meet some of the priorities defined in the 2030 Agenda for Sustainable Development [50].

Age: In both genders, especially women, increasing age contributed to a higher CN, which is in line with other studies [8,10,48,51]. According to the literature, the higher CN in older individuals is linked to a higher number of visits to natural spaces [52] and to greater opportunities for engaging with them [53]. Older people develop deeper connections with their surroundings, attaching greater meaning to the little things in life [54].

Occupational status: The study revealed a significant association between occupational status and CN. Some authors point towards a greater connection to the natural environment in retired [55] or unemployed individuals [49]. According to Freeman et al. [56], in older adults, retirement can provide more time and increased opportunities to engage with nature. Similarly, in unemployed individuals, this higher connection may be related to the fact that they have more time to experience nature, contributing to the development of a stronger affective connection with the natural environment. As stated in Hartig et al. [57], most employees work in confined spaces (e.g., offices, factories, shopping centres), away from natural ecosystems, which may result in a less affective connection with the natural environment for this occupation status.

The number of children: Women with more children showed a higher CN. In the literature, we did not identify any studies analysing the relationship between these variables, but some authors [58,59] document a positive association between motherhood and pro-environmental behaviours, expressed in sustainability and environmental protection actions. They may have eventually mediated this relationship, but it is not possible to prove this inference from our results. This study identified a significant correlation between CN and VFA, with the latter variable being directly related to maternity. In every pregnancy, adiposity is preferentially deposited in the visceral adipose tissue, especially during the third trimester of gestation [60], with the gain being slower in overweight/obese women compared with women of normal weight [61].

Dog ownership: In the present investigation, men with dogs exhibited a higher CN. These results were in agreement with other studies looking at the two genders together [62,63,64]. The same authors justified this relationship using the feelings of belonging instilled by pet ownership, which is reflected in the greater attachment to the natural environment. This association was only identified in males, perhaps because men spend more time taking care of their dogs compared to women and are less involved in completing household and/or parental tasks [65,66].

Residence place: Many studies [67,68,69] demonstrated that individuals who live in urban areas document less CN compared with those who live in rural areas. In the present investigation, and despite the extent of green space in the sample area of residence (19,204 ha), no associations were identified between these two variables. According to White et al. [70], more than living in green/coastal areas, it is recreational visits to these natural environments that translate into greater community benefits and perhaps more of an affectionate relationship with nature.

### 4.2. Connectedness to Nature and Physical Activity

Women with a greater CN performed a greater number of daily steps. As reported by Martin et al. [71], a greater connection to nature can contribute to a greater contact with it, leading individuals to choose green spaces to live in or to visit these environments more often. As is recognized in the literature [72,73,74], these spaces are excellent places for physical activity and encourage more active lifestyles, such as taking a greater number of daily steps. Additionally, in our study, women with higher levels of visceral adiposity also completed a greater number of daily steps and showed a greater affective connection with nature. For some authors [75,76], individuals with high levels of adiposity were shown to privilege walking for PA. The VFA may possibly have mediated the relationship between CN and the number of steps/day; however, we cannot prove this inference from our results with complete certainty.

Along these lines, the definition of environmental practices and policies that encourage contact and CN, translated into interventions such as park prescriptions or guidelines for exposure to nature and urban planning, is essential in human health and the sustainability of natural ecosystems [71,77,78].

### 4.3. Connectedness to Nature and Body Composition

In the present research, women with higher levels of central adiposity and a more compromised muscular condition revealed a higher CN. Several aspects may be related to the results. First, an increased deposition of triglycerides in intra-abdominal adipocytes and reduced muscle mass tends to become more pronounced in women with aging, particularly with o depletion due to menopause [79], and there is a positive association between age and connection to the natural environment.

### 4.4. Variation in Physical Activity and Body Composition Parameters Correlated with Connectedness to Nature

In the female sample, adiposity levels and muscle condition influenced the variation in MVPA and the number of daily steps. This relationship could partly be explained by their average age (40 years) and the fact that 34% of the women were in menopause transition or postmenopausal. According to Juppi et al. [80], menopausal transition is a critical factor in the loss of muscle mass, with a marked decrease in the size of type II muscle fibres, while oxidative fibres remain relatively unchanged and there is no change in their cross-sectional area. The hormonal changes that occur in this climacteric stage (reduction of oestrogen, growth hormone, and insulin-like growth factor type 1) are the main causes of this muscle compromise [81], particularly affecting the lower limbs [82]. This is reflected by a decrease in ASMMI and adversely affects MVPA levels in women.

In the present study, the preservation of muscle mass in the upper and lower limbs contributed to increased levels of MVPA in women. The results are in agreement with those identified by other authors [83]. This relationship, which is identified only in women, may be associated with their training preference for the appendicular musculature, particularly the lower region [84].

As in previous studies, our study showed an inverse relationship between %FM and MVPA [85,86,87]. It is acknowledged in the literature that total adiposity peaks are reached in both genders after 50 years [88], affecting especially women, due to menopause [89]. Our female sample in this age group presented mostly with obesity (73.2%), with about 34% of women being in stages of reproductive aging that generate exponential increases in adiposity levels, reducing the usual levels of PA and compromising their quality of life [89].

The occupational status explained the variation in the number of daily steps for men, which was higher in employees. For some authors [90,91], the number of daily steps differs by occupation, with delivery workers and those working in the retail or service industry exhibiting less sedentary behaviour compared with those who spend several hours sitting at their jobs. Similarly, employed people may have better financial resources to support participation in sports and recreational activities [92] and are more likely to live in areas that promote active mobility. In our study, we cannot explain why this relationship was identified only in the male gender; this is probably the result of the types of occupation typical for men, and we recognize that it would have been useful to collect this information.

In this investigation, older women performed a greater number of steps, with the results being similar to those documented by other authors [93,94,95]. The preference for walking by older individuals is justified by the fact that this activity requires less confidence in the body’s capacity than most other forms of physical activity [96].

Recognizing that the female gender faces significant inequalities in access to health (education, income, employment, etc.), greater barriers to engaging in PA and greater spending on health care expenses [97], the availability of green spaces proves to be very important in meeting the recommended daily PA guidelines [39] and controlling several health conditions that particularly affect adult and older women, such as obesity, depression and anxiety [98]. Our results thus highlight the importance of creating healthy and sustainable cities that positively influence all population groups, particularly women and older people. Given that the population is ageing and that, by 2030, over 80% of the European population will live in urban areas, cities play a key role in promoting health and well-being, and there is a growing need to develop ways to support participation in PA and reduce sedentary behaviour [99].

Women with better muscular condition in the trunk have a higher number of daily steps, a result that can be explained by the fact that the muscles of this region play a key role in the movement and stabilization of the upper body during sports activities [100]. The trunk muscles (deep and superficial) play a key role in stabilizing the lumbo-pelvic region, improving gait [101]. It should also be noted that isometric and dynamic exercises at the trunk level contribute to the improvement in walking patterns, benefiting balance and stability [102]. Therefore, it is expected that a stronger central region would translate into higher levels of PA, particularly the performance of a greater number of daily steps.

It was also women with higher levels of intra-abdominal adiposity who performed a greater number of daily steps, results that are contradictory to those found in the literature. According to several authors [103,104,105,106], women who perform more steps/day (≥10,000) tend to exhibit a lower VFA. In our study, this relationship was positive, perhaps since women with high levels of central adiposity may exhibit a preference for walking. Similar results were identified in the systematic review conducted by Baillot et al. [107], which could be because this activity does not require any specific skill, equipment or place and can be easily integrated into everyday life [108]. According to the first author [107], regular walking is often proposed by health professionals as an option in the management of obesity and inactivity.

In both genders, the visceral fat area was explained by age and relative fat mass. The literature documents that, independent of weight variations, the %FM significantly increases with advancing age [109,110], and this aggravation is particularly pronounced with oestrogen depletion [89].

The regression that developed for VFA in women also revealed that employed female individuals had higher levels of central adiposity. Considering that, in our sample, women exhibited higher educational levels, it is likely that this can be related to the performance of more sedentary professional functions and reduced leisure time [91]. In the present investigation, women with higher levels of central adiposity were those who showed a preference for gyms for PA practice. Our results are important for health and fitness specialists working in public spaces, particularly in facilities such as gyms and fitness centres, which in recent decades have increasingly emphasized a holistic approach to health and fitness, of which the loss of fat mass is a part [111]. It is necessary for these activity centres to provide a welcoming environment that limits stigmatizing situations, ensuring that people of all body types can be motivated to exercise, free of direct and indirect forms of judgment [112].

The number of children also explained the variation in men’s intra-abdominal adiposity levels, which were higher in those who had more children. The majority of studies have focused only on women, due to the assumption that there is a biological pathway between pregnancy, childbirth and obesity. In this regard, several studies document an association between parity and visceral fat in women [113,114,115], contrary to the opposite gender, where results are still unclear [116,117].

Regarding the regressions that developed for total and regional muscle conditions, we found that the presence of a higher amount of relative fat mass negatively influences SMM in both genders, with the regressions showing very similar standardized coefficients. The presence of hypertrophied adipocytes and the infiltration of macrophages in the adipose tissue generates the altered production of adipokines (reduced adiponectin and increased leptin, interleukin 6 and tumour necrosis factor-alpha), which translates into an inflammatory process at the muscle level and stimulates the infiltration of fat into the muscle [118].

In both genders, FFM was selected as a predictor in the models developed for muscle condition. Since FFM comprises the internal organs, amount of water and skeletal muscle mass, it is expected that higher levels of FFM translate into a better musculoskeletal condition [119]. Our results highlight the importance of preserving body composition components in adulthood, particularly fat-free mass, by attenuating the age-related changes.

The presence of higher levels of MVPA in women had a positive influence on their muscle condition. In fact, a growing number of studies have provided strong recommendations for PA as the primary treatment for loss of muscle mass [120,121]. According to Mendoza et al. [122], the practice of moderate PA, especially from the fourth decade in women, can benefit their health, attenuating gains in adiposity and the loss of muscle and bone mass.

Age negatively influences appendicular muscle mass in both genders, with a higher standardized coefficient in men. The greater loss of ASMM in the latter is explained by the fact that the trunk region shows a high number of testosterone receptors [123] and that training this body region is usually valued by men [124,125], favouring a mesomorphic somatotype, which improves performance in strength, endurance and coordination tests and is more attractive to the female gender [126,127].

## 5. Strengths and Limitations of the Study

The current study had several strengths, including the assessment of PA levels by accelerometery and the inclusion of a range of sociodemographic and body composition variables. Likewise, the analysis being separately conducted for each gender was revealed to be very important.

Despite the previously explained strengths, our study contains some limitations that should be addressed in future work. First, our sample included few older adults, and it would have been pertinent to include more individuals aged 65 and older. Our research would also have benefited if we had included a larger number of males. Second, although the validity of InBody 720 is documented in the literature [128,129,130,131,132], bioimpedance is not considered as a reference method to assess body composition [130,131]. Third, accelerometers do not accurately record some activities, such as cycling or weight training (due to the lack of up-and-down motion), and participants were told to remove them while doing any water sports. Furthermore, the results obtained by accelerometry could be subject to bias, since the study participants knew that their movements were being monitored, and thus might have behaved differently than they would if they were not being observed. The time of year when the data were collected not only influences physical activity levels, but also the connection with nature itself, reflecting different levels of physical comfort, weather patterns, or a differentiated state of natural spaces associated with seasonality. Finally, since the scale used to measure the CN only assesses the affective component, we believe that it would be useful to apply a scale that assesses other dimensions, such as the cognitive and behavioural components.

## 6. Future Research

Our results provide useful information for guiding future research regarding the promotion of PA levels and the improvement of body composition. The analysis of the demographic variables proved to be of considerable importance, confirming the need for their inclusion in future investigations. It is equally important to conduct longitudinal studies that examine the association of CN with PA and body composition. Quasi-experimental studies and randomized trials may provide more detail on how nature influences health. The use of more precise methodologies to assess body composition and the inclusion of more representative samples from different geographical areas, occupations and age groups is essential if we are to better understand the contexts and periods where connection to the natural environment could have the strongest impact on an individual’s health. Furthermore, the combination of accelerometery with GPS data would have allowed objective and high temporal and spatial resolution measurements of the environments in which PA was taking place. The analysis of interventions designed to promote PA levels and body composition through engagement with the natural environment would also be welcome.

## 7. Conclusions

To the best of our knowledge, our study is the first to investigate the relationship between connectedness with nature and body composition. Contrary to our intuition, this connection to the natural environment did not explain the variation in PA and body composition in both genders, despite the significant associations that have been identified between CN and daily steps, central adiposity and muscle condition in women.

Overall, the results of the present study indicated that the increase in % fat mass explained the higher levels of central adiposity and the reduction in SMM in both genders, with the latter being improved with the practice of higher MVPA in women. Additionally, we found that demographic variables did not have any explanatory power in the variation in SMM and ASMM in both genders.

## Figures and Tables

**Table 1:** Baseline characteristics of study participants.

Variables	Overall (*n* = 219) Mean ± SD	Men (*n* = 77) Mean ± SD	Women (*n* = 142) Mean ± SD	*p*
Age (years)	40.63 ± 15.35	41.62 ± 15.12	40.10 ± 15.49	0.60 ^(b)^
Anthropometry/Body composition				
Body mass (kg)	67.07 ± 11.95	76.96 ± 10.61	61.70 ± 8.82	**<0.01 ^(b)^**
Body height (m)	1.67 ± 0.10	1.77 ± 0.07	1.62 ± 0.06	**<0.01 ^(a)^**
Body mass index (kg/m^2^)	23.88 ± 3.30	24.61 ± 3.05	23.49 ± 3.37	**0.02 ^(a)^**
Fat mass (kg)	18.14 ± 7.10	15.56 ± 6.60	19.53 ± 6.99	**<0.01 ^(b)^**
Fat mass (%)	26.98 ± 8.99	19.83 ± 6.56	30.85 ± 7.67	**<0.01 ^(a)^**
Visceral fat area (cm^2^)	86.52 ± 39.49	89.25 ± 44.38	85.03 ± 36.65	0.54 ^(b)^
Fat-free mass (kg)	49.02 ± 11.09	61.40 ± 7.77	42.31 ± 5.34	**<0.01 ^(a)^**
Skeletal muscle mass (kg)	27.21 ± 6.77	34.79 ± 4.72	23.10 ± 3.23	**<0.01 ^(a)^**
Appendicular skeletal muscle mass (kg)	20.27 ± 5.17	26.04 ± 3.49	17.15 ± 2.59	**<0.01 ^(a)^**
Appendicular skeletal muscle mass index (kg/m^2^)	7.13 ± 1.09	8.29 ± 0.66	6.49 ± 0.68	**<0.01 ^(a)^**
Trunk skeletal muscle mass (kg)	21.96 ± 4.83	27.24 ± 3.53	19.09 ± 2.39	**<0.01 ^(a)^**
Trunk skeletal muscle mass index (kg/m^2^)	7.76 ± 1.06	8.70 ± 0.88	7.25 ± 0.76	**<0.01 ^(a)^**
Accelerometer data				
TPA (min/week)	705.64 ± 297.07	673.88 ± 272.91	722.87 ± 308.95	0.31 ^(b)^
MVPA (min/week)	179.13 ± 114.36	187.29 ± 110.46	174.71 ± 116.57	0.29 ^(b)^
Steps/day (No.)	12,924.96 ± 3769.01	13,521.99 ± 3825.42	12,601.22 ± 3711.48	0.12 ^(b)^
Connectedness to nature (points)	3.74 ± 0.43	3.72 ± 0.44	3.75 ± 0.42	0.59 ^(a)^

Abbreviations: Standard Deviation (SD), ^(a)^ *t*-test for independent samples, ^(b)^ Mann-Whitney test, TPA = Total Physical Activity, MVPA = Moderate-Vigorous Physical Activity.

**Table 2:** Demographic data documented by participants.

Variables	Overall (*n* = 219) *n* (%)	Men (*n* = 77) *n* (%)	Women (*n* = 142) *n* (%)
Marital status			
Married/cohabiting	106 (48.4)	44 (57.1)	62 (43.7)
Single	98 (44.7)	31 (40.3)	67 (47.2)
Separated/divorced/widowed	15 (6.8)	2 (2.6)	13 (9.2)
Number of children			
0	103 (47.0)	34 (44.2)	69 (48.6)
1	42 (19.2)	11 (14.3)	31 (21.8)
2	62 (28.3)	28 (36.4)	34 (23.9)
≥3	12 (5.5)	4 (5.2)	8 (5.6)
Education			
No education	1 (0.5)	-	1 (0.7)
Elementary school	7 (3.2)	3 (3,9)	4 (2.8)
Middle school	12 (5.5)	6 (7.8)	6 (4.2)
High school	86 (39.3)	28 (36.4)	58 (40.8)
University	41 (18.7)	16 (20.8)	25 (17.6)
Post-graduate/master’s/doctorate	72 (32.9)	24 (31.2)	48 (33.8)
Occupational status			
Employed	147 (67.1)	55 (71.4)	92 (64.8)
Unemployed	4 (1.6)	2 (2.6)	2 (1.4)
Retired	5 (2.3)	1 (1.3)	4 (2.8)
Student	55 (25.1)	17 (22.1)	38 (26.8)
Other	8 (3.7)	2 (2.6)	6 (4.2)
Residence place			
Rural	59 (26.9)	23 (29.9)	36 (25.4)
Semi-urban	37 (16.9)	6 (7.8)	31 (21.8)
Urban	123 (56.2)	48 (62.3)	75 (52.8)
Car ownership			
No	24 (11.0)	7 (9.1)	17 (12.0)
Yes	195 (89.0)	70 (90.9)	125 (88.0)
Dog ownership			
No	148 (67.6)	50 (64.9)	98 (69.0)
Yes	71 (32.4)	27 (35.1)	44 (31.0)
Contexts for PA			
In parks, outdoors, etc.	120 (54.8)	46 (59.7)	74 (52.1)
At home	12 (5.5)	2 (2.6)	10 (7.0)
On the way between home and school, work or shop	4 (1.8)	-	4 (2.8)
At a health or fitness centre	65 (29.7)	21 (27.3)	44 (31.0)
At a sport centre	3 (1.4)	2 (2.6)	1 (0.7)
At work	-	-	-
At school or university	1 (0.5)	1 (1.3)	-
Elsewhere (spontaneous)	13 (5.9)	5 (6.5)	8 (5.6)
Don’t know	1 (0.5)	-	1 (0.7)

Abbreviations: PA = Physical Activity.

**Table 3:** Association between connectedness to nature with PA, body composition and demographic variables.

Variables	Connectedness to Nature	
	Men (*n* = 77)	Women (*n* = 142)
TPA (min/week)	0.090	0.102
MVPA (min/week)	0.074	−0.015
Steps/day (No.)	0.169	0.209 *
Contexts for PA η^2^	0.330	0.285
Fat mass (kg)	0.158	0.071
Fat mass (%)	0.202	0.137
Visceral fat area (cm^2^)	0.216	0.274 **
Fat-free mass (kg)	−0.110	−0.163
Skeletal muscle mass (kg)	−0.112	−0.165 *
Appendicular skeletal muscle mass (kg)	−0.163	−0.187 *
Appendicular skeletal muscle mass index (kg/m^2^)	−0.047	−0.059
Trunk skeletal muscle mass index (kg/m^2^)	0.133	0.067
Age	0.245 *	0.384 **
Marital status η^2^	0.292 *	0.381 **
Number of children	0.082	0.235 **
Education r*_s_*	0.256	0.257
Occupational status η^2^	0.433 **	0.325 **
Residence place η^2^	0.269	0.197
Car ownership r*_pb_*	0.091	0.105
Dog ownership r*_pb_*	0.329 **	−0.048

Abbreviations: PA = Physical Activity, TPA = Total Physical Activity, MVPA = Moderate-Vigorous Physical Activity, η^2^ = Eta square, r*_s_* = Spearman’s correlation coefficient, r*_pb_* = point–biserial correlation coefficient, * *p* ≤ 0.05, ** *p* ≤ 0.01.

**Table 4:** Correlations of PA with body composition in both genders.

Variables	TPA		MVPA		Steps/Day		Contexts for PA η^2^	
	Men	Women	Men	Women	Men	Women	Men	Women
Fat mass (%)	−0.134	−0.112	−0.143	−0.263 **	−0.043	−0.015	0.273	0.158
Visceral fat area (cm^2^)	−0.114	0.028	−0.190	−0.226 **	0.009	0.205 *	0.377	0.296 *
Fat-free mass (kg)	0.153	0.103	0.131	0.162	−0.046	0.027	0.344	0.313
Skeletal muscle mass (kg)	0.149	0.121	0.130	0.180 *	−0.060	0.041	0.341	0.320
Appendicular skeletal muscle mass (kg)	0.116	0.062	0.142	0.132	−0.070	−0.017	0.285	0.283
Appendicular skeletal muscle mass index (kg/m^2^)	0.175	0.209 *	0.143	0.185 *	−0.012	0.205 *	0.354	0.313
Trunk skeletal muscle mass index (kg/m^2^)	0.263 *	0.269 **	0.086	0.152	0.141	0.324 **	0.441 **	0.329 *

Abbreviations: PA = Physical Activity, TPA = Total Physical Activity, MVPA = Moderate-Vigorous Physical Activity, η2 = Eta square, * *p* ≤ 0.05, ** *p* ≤ 0.01.

**Table 5:** Multiple linear regressions developed in both genders for the PA.

**MVPA**						
Men (*n* = 77)						
Model	b	95% CI b	β	*p*	Adjusted R^2^	SEE
Predictors: -						
Women (*n* = 142) δ						
Constant	2.502	(2.290, 2.714)	-	<0.01	0.074	0.307
Fat mass (%)	−0.012	(−0.018, −0.005)	−0.283	<0.01		
Predictors: age, fat mass (%), visceral fat area, skeletal muscle mass and appendicular skeletal muscle mass index						
**Steps/Day**						
Men (*n* = 77) δ						
Model	b	95% CI b	β	*p*	Adjusted R^2^	SEE
Constant	4.050	(0.014,4.100)		<0.01	0.098	0.116
Occupational status: employee	0.089	(0.031, 0.148)	0.332	<0.01		
Predictors: age, number of children, occupational status and dog ownership						
Women (*n* = 142)						
Constant	−1725.730	(−7319.511, 3868.052)	-	0.543	0.204	3312.197
Age	130.433	(71.711, 189.154)	0.544	<0.01		
Trunk skeletal muscle mass index	1688.422	(882.089, 2494.756)	0.345	<0.01		
Visceral fat area	−36.987	(−63.391, −10.584)	−0.365	0.006		
Predictors: CN, age, marital status, number of children, occupational status, visceral fat area, appendicular skeletal muscle mass index and trunk skeletal muscle mass index						

Abbreviations: b = non-standardized coefficients, β = standardized coefficients, CI = Confidence Interval, Adjusted R^2^ = adjusted coefficient of determination, SEE = Standard Error Estimate, δ = Distribution standardization through a logarithmic transformation of the variable, MVPA = Moderate-vigorous Physical Activity, CN = Connectedness to Nature.

**Table 6:** Multiple linear regressions developed in both genders for body composition.

**Visceral Fat Area**						
Men (*n* = 77)						
Model	b	95% CI b	β	*p*	Adjusted R^2^	SEE
Constant	−43.367	(−55.288, −31.446)	-	<0.01	0.908	13.484
Fat mass (%)	5.296	(4.753, 5.839)	0.782	<0.01		
Age	0.519	(0.176, 0.863)	0.177	0.004		
Number of children	5.835	(1.061, 10.609)	0.133	0.017		
Predictors: age, marital status, number of children, occupational status, car ownership, and fat mass (%)						
Women (*n* = 142)						
Constant	57.076	(−65.269, −48.883)	-	<0.01	0.911	10.961
Fat mass (%)	2.887	(2.618, 3.155)	0.604	<0.01		
Age	1.187	(1.024, 1.350)	0.502	<0.01		
Occupational status: employee	6.197	([1.392, 11.001)	0.081	0.012		
Contexts for PA: health or fitness centre	4.691	(0.670, 8.712)	0.059	0.023		
Predictors: CN, age, marital status, number of children, occupational status, car ownership, MVPA, steps/day, contexts for PA and fat mass (%)						
**Skeletal Muscle Mass**						
Men (*n* = 77)						
Model	b	95% CI b	β	*p*	Adjusted R^2^	SEE
Constant	−1.968	(−2.657, −1.280)	-	<0.01	0.995	0.324
Fat-free mass	0.603	(0.593, 0.613)	0.993	<0.01		
Fat mass (%)	−0.014	(−0.026, −0.003)	−0.020	0.017		
Predictors: Fat mass (%) and fat-free mass						
Women (*n* = 142)						
Constant	−2.089	(−2.470, −1.707)	-	<0.01		
Fat-free mass	0.598	(0.591, 0.605)	0.990	<0.01		
MVPA	0.000	(0.000, 0.001)	0.016	0.009	0.996	0.216
Fat mass (%)	−0.007	(−0.012, −0.002	−0.016	0.011		
Predictors: CN, age, education, MVPA, fat mass (%) and fat-free mass						
**Appendicular Skeletal Muscle Mass**						
Men (*n* = 77)						
Model	b	95% CI b	β	*p*	Adjusted R^2^	SEE
Constant	0.673	(−1.353, 2.700)	-	0.510	0.930	0.925
Fat-free mass	0.424	(0.395, 0.452)	0.943	0.000		
Age	−0.016	(−0.030, −0.001)	−0.068	0.035		
Predictors: age, fat mass (%) and fat-free mass						
Women (*n* = 142)						
Constant	−2.406	(−3.266, −1.546)	-	<0.01	0.952	0.570
Fat-free mass	0.469	(0.420, 0.487)	0.967	<0.01		
Age	−0.007	(−0.013, 0.000)	−0.040	0.035		
Predictors: CN, age, education, fat mass (%) and fat-free mass						

Abbreviations: b = non-standardized coefficients, β = standardized coefficients, CI = Confidence Interval, Adjusted R^2^ = adjusted coefficient of determination, SEE = Standard Error Estimate, MVPA = Moderate-Vigorous Physical Activity, CN = Connectedness to Nature.

## Data Availability

The data presented in this study are available on request from the corresponding author. The data are not publicly available.

## Supplementary Materials

The following are available online at https://www.mdpi.com/article/10.3390/ijerph182211951/s1, Figure S1: Land cover classification of the study area; Table S1: Operationalization of demographic variables; Table S2: Association of demographic data with age, physical activity and body composition and Table S3: Correlations developed for the body composition variables in both genders.
