# Supplementary material for: Connectedness to Nature Does Not Explain the Variation in Physical Activity and Body Composition in Adults and Older People
Source: Int J Environ Res Public Health. 2021 Nov 14;18(22):11951. doi: 10.3390/ijerph182211951 (PMC8619523; doi:10.3390/ijerph182211951)
Supplement: Supplementary file 1 [file ijerph-18-11951-s001.zip › Supplementary Data.pdf]

Supplementary Data

Table S1 - Operationalisation of demographic variables

	Measure	Item	Responses
Demographic variables	Age	What was your age last birthday?	
	Gender	Sex of respondent?	Male (0); Female (1)
	Marital status	Marital status of respondent	Married/cohabiting (1); Single (2); Separated/divorced/widowed (3)
	Number of children	How many children do you have?	0; 1; 2; ≥ 3
	Education	What are your academic qualifications?	No Education (0); Elementary school (1); Middle school (2); High school (3); University (4); Post-graduation/master's/doctorate (5)
	Occupational status	What is your occupational situation?	Employee (1); Unemployed (2); Retired (3); Student (4); Other (5)
	Residence place	What is your place of residence?	Rural (1); Semi-urban (2); Urban (3)
	Car ownership	Do you own or have access to a car?	No (0); Yes (1)
	Dog ownership	Do you have dogs that you usually walk?	No (0); Yes (1)
	**Contexts for physical activity**	**What is your favorite/privileged places for physical activity?**	**In parks, outdoors, etc. (1); At home (2); On the way between home and school, work or shop (3); At a health or fitness centre (4); At a sports centre (5); At work (6); At school or university (7); Elsewhere (spontaneous) (8); or Don´t know (9)**

Table S2. Association of demographic data with age, physical activity and body composition.

Variables	Age	Marital status	Number of children	Education	Occupational status	Residence place	Car ownership	Dog ownership
MEN (n=77)								
Age (years)		0.866** η^2	0.770** r	0.095 r_s	0.729** η^2	0.060 η^2	0.317** r_{pb}	-0.015 r_{pb}
Total PA (TPA, min/week)	-0.032 r	0.059 η^2	0.113 r	0.065 r_s	0.318 η^2	0.162 η^2	0.073 r_{pb}	0.275* r_{pb}
Moderate-vigorous PA (MVPA, min/week)	0.044 r	0.103 η^2	-0.015 r	0.068 r_s	0.128 η^2	0.079 η^2	-0.022 r_{pb}	0.197 r_{pb}
Steps/day (nº)	0.250* r	0.242 η^2	0.268* r	0.049 r_s	0.380* η^2	0.189 η^2	0.116 r_{pb}	0.255* r_{pb}
Contexts for PA (1-8)	0.405* η^2	0.264 V	0.270 η^2	0.321* V	0.235 V	0.262 V	0.378 V	0.249 V
Fat mass (FM, %)	0.499** r	0.353** η^2	0.364** r	-0.044 r_s	0.485** η^2	0.251 η^2	0.291* r_{pb}	-0.101 r_{pb}
Visceral fat area (VFA, cm^2)	0.670** r	0.553** η^2	0.554** r	0.025 r_s	0.580** η^2	0.201 η^2	0.310** r_{pb}	-0.083 r_{pb}
Fat-free mass (FFM, kg)	-0.295** r	0.165 η^2	-0.075 r	-0.005 r_s	0.209 η^2	0.003 η^2	-0.018 r_{pb}	-0.086 r_{pb}
Skeletal muscle mass (SMM, kg)	-0.321 r	0.183 η^2	-0.100 r	-0.013 r_s	0.226 η^2	0.016 η^2	-0.024 r_{pb}	-0.094 r_{pb}
Appendicular skeletal muscle mass (ASMM, kg)	-0.347** r	0.227 η^2	-0.095 r	-0.021 r_s	0.235 η^2	0.038 η^2	-0.103 r_{pb}	-0.086 r_{pb}
Appendicular skeletal muscle mass index (ASMMI, kg/m^2)	-0.194 r	0.081 η^2	0.011 r	-0.080 r_s	0.174 η^2	0.039 η^2	0.087 r_{pb}	-0.065 r_{pb}
Trunk skeletal muscle mass index (TSMMI, kg/m^2)	0.025 r	0.157 η^2	0.084 r	-0.093 r_s	0.278 η^2	0.064 η^2	0.201 r_{pb}	0.014 r_{pb}
WOMEN (n=142)								
Age (years)		0.767** η^2	0.698 r	0.186* r_s	0.784** η^2	0.079 η^2	0.255** r_{pb}	-0.169* r_{pb}
Total PA (TPA, min/week)	0.108 r	0.212** η^2	0.230** r	-0.079 r_s	0.230* η^2	0.186 η^2	0.040 r_{pb}	0.104 r_{pb}
Moderate-vigorous PA (MVPA, min/week)	-0.177* r	0.185 η^2	-0.012 r	-0.077 r_s	0.155 η^2	0.193 η^2	-0.067 r_{pb}	0.125 r_{pb}
Steps/day (nº)	0.337** r	0.291** η^2	0.322** r	-0.071 r_s	0.362** η^2	0.143 η^2	0.062 r_{pb}	0.021 r_{pb}
Contexts for PA (1-8)	0.992 η^2	0.272* V	0.245 η^2	0.235 V	0.242 V	0.191 V	0.274 V	0.221 V
Fat mass (FM, %)	0.413** r	0.318** η^2	0.243** r	-0.055 r_s	0.246 η^2	0.030 η^2	0.102 r_{pb}	-0.034 r_{pb}
Visceral fat area (VFA, cm^2)	0.790** r	0.573** η^2	0.541** r	0.098 r_s	0.596** η^2	0.054 η^2	0.190* r_{pb}	-0.077 r_{pb}
Fat-free mass (FFM, kg)	-0.204* r	0.130 η^2	-0.109 r	0.225** r_s	0.101 η^2	0.056 η^2	-0.062 r_{pb}	0.049 r_{pb}
Skeletal muscle mass (SMM, kg)	-0.218** r	0.147 η^2	-0.115 r	0.214* r_s	0.119 η^2	0.052 η^2	-0.071 r_{pb}	0.054 r_{pb}
Appendicular skeletal muscle mass (ASMM, kg)	-0.238** r	0.161 η^2	-0.142 r	0.228** r_s	0.114 η^2	0.070 η^2	-0.056 r_{pb}	0.055 r_{pb}
Appendicular skeletal muscle mass index (ASMMI, kg/m^2)	0.006 r	0.020 η^2	0.014 r	0.185* r_s	0.142 η^2	0.026 η^2	-0.002 r_{pb}	0.000 r_{pb}
Trunk skeletal muscle mass index (TSMMI, kg/m^2)	0.236** r	0.148 η^2	0.164 r	0.061 r_s	0.288* η^2	0.048 η^2	0.052 r_{pb}	-0.013 r_{pb}

Abbreviations: PA = Physical activity, η^2 = Eta square, r_s = Spearman's correlation coefficient, r_{pb} = point–biserial correlation coefficient, r = Peasorn correlation coefficient, V = Cramér's V coefficient, * $p \leq 0.05$, ** $p \leq 0.01$

Table S3 - Correlations developed for the body composition variables in both genders

	FM (kg)	FM (%)	VFA (cm^2)	FFM (kg)	SMM (kg)	ASMM (kg)	ASMMI (kg/m^2)	TSMMI (kg/m^2)
FM (kg)		**0.946****	**0.898****	0.085	0.067	0.032	0.204	**0.282***
FM (%)	**0.904****		**0.919****	-0.209	**-0.227***	**-0.260***	-0.069	0.091
VFA (cm^2)	**0.795****	**0.809****		-0.048	-0.070	-0.088	0.064	0.206
FFM (kg)	0.042	**-0.302****	-0.096		**0.998****	**0.962****	**0.682****	**0.920****
SMM (kg)	0.026	**-0.319****	-0.109	**0.998****		**0.961****	**0.691****	**0.920****
ASMM (kg)	0.068	**-0.272****	-0.124	**0.975****	**0.968****		0.855	**0.504****
ASMMI, (kg/m^2)	**0.222****	-0.099	0.105	**0.872****	**0.879****	**0.859****		**0.812****
TSMMI (kg/m^2)	**0.381****	0.137	**0.407****	**0.615****	**0.631****	**0.534****	**0.851****	

Notes: above the diagonal line the values refer to men; below the diagonal line the values refer to women; Abbreviations: Fat Mass (FM, kg), Fat Mass (FM, %), Visceral Fat area (VFA), Fat-Free Mass (FFM), Skeletal Muscle Mass (SMM), Appendicular Skeletal Muscle Mass (ASMM), Appendicular Skeletal Muscle Mass Index (ASMMI), Trunk Skeletal Muscle Mass Index (TSMMI), * $p \leq 0.05$, ** $p \leq 0.01$
