# Supplementary figures and images for: Connectedness to Nature Does Not Explain the Variation in Physical Activity and Body Composition in Adults and Older People
Source: Int J Environ Res Public Health. 2021 Nov 14;18(22):11951. doi: 10.3390/ijerph182211951 (PMC8619523; doi:10.3390/ijerph182211951)

Supplement Figure S1. Land cover classification of the study area

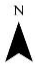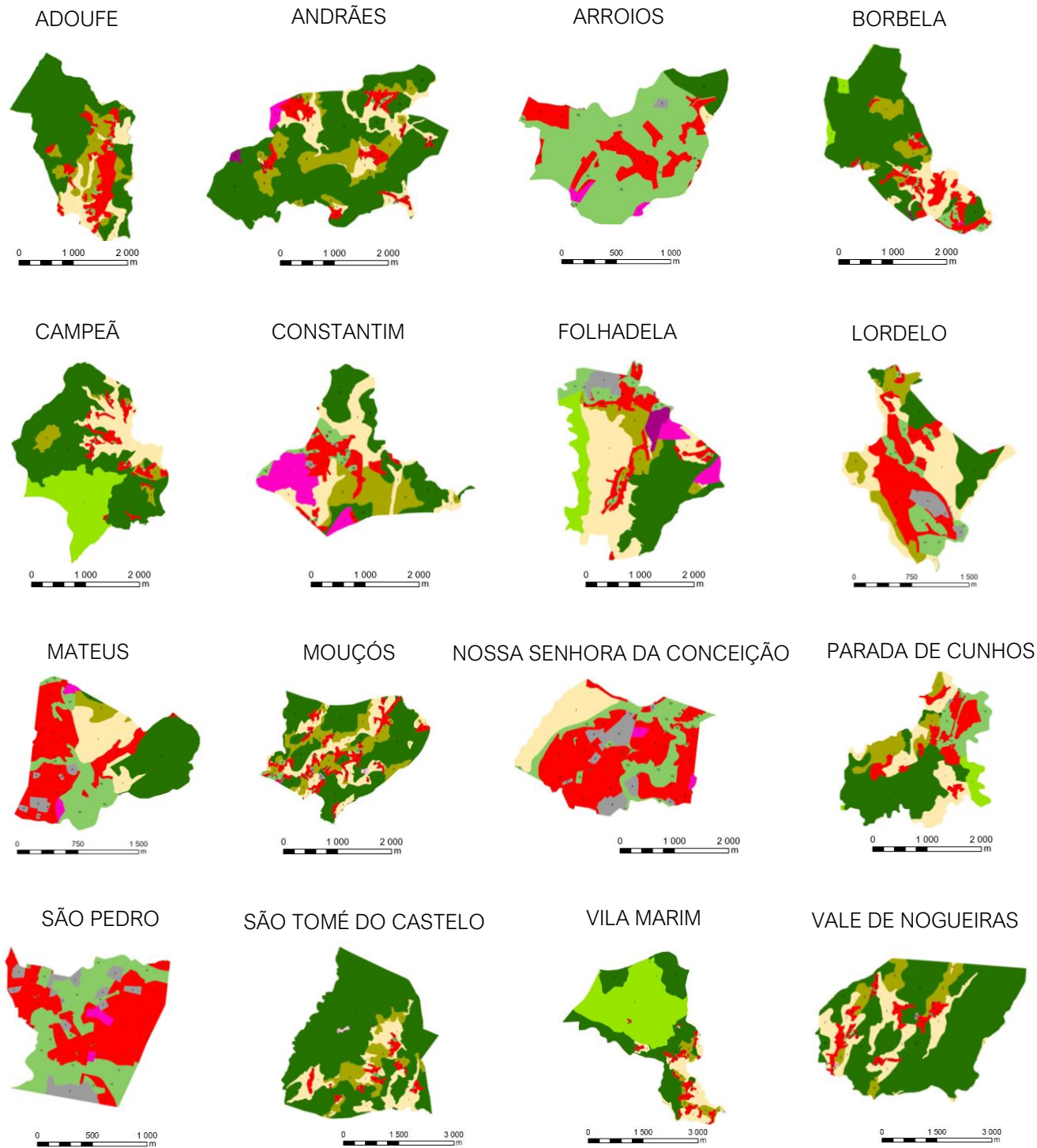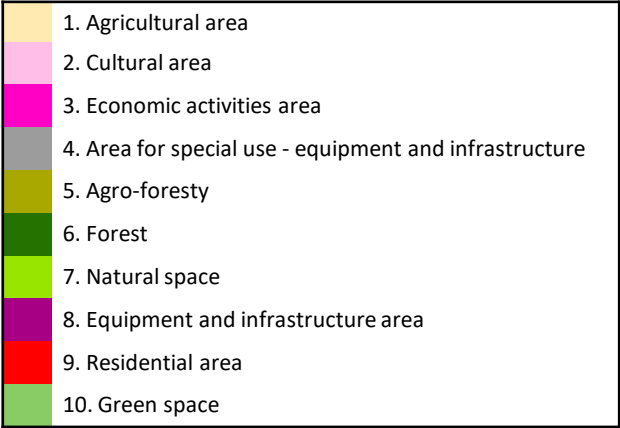

Supplement: Supplementary file 1 [file ijerph-18-11951-s001.zip › Supplement 1.pdf]
